# Face scanning and spontaneous emotion preference in Cornelia de Lange syndrome and Rubinstein-Taybi syndrome

**DOI:** 10.1186/s11689-015-9119-4

**Published:** 2015-07-30

**Authors:** Hayley Crawford, Joanna Moss, Joseph P. McCleery, Giles M. Anderson, Chris Oliver

**Affiliations:** Centre for Research in Psychology, Behaviour and Achievement, Coventry University, Coventry, CV1 5FB UK; Cerebra Centre for Neurodevelopmental Disorders, School of Psychology, University of Birmingham, Edgbaston, B15 2TT UK; Institute of Cognitive Neuroscience, University College London, 17 Queen Square, London, WC1N 3AR UK; Center for Autism Research, Children’s Hospital of Philadelphia, 3535 Market Street, Philadelphia, PA 19104 USA; School of Psychology, Oxford Brookes University, Headington Campus, Oxford, OX3 0BP UK

**Keywords:** Cornelia de Lange syndrome, Rubinstein-Taybi syndrome, Eye-tracking, Emotion preference, Eye gaze

## Abstract

**Background:**

Existing literature suggests differences in face scanning in individuals with different socio-behavioural characteristics. Cornelia de Lange syndrome (CdLS) and Rubinstein-Taybi syndrome (RTS) are two genetically defined neurodevelopmental disorders with unique profiles of social behaviour.

**Methods:**

Here, we examine eye gaze to the eye and mouth regions of neutrally expressive faces, as well as the spontaneous visual preference for happy and disgusted facial expressions compared to neutral faces, in individuals with CdLS versus RTS.

**Results:**

Results indicate that the amount of time spent looking at the eye and mouth regions of faces was similar in 15 individuals with CdLS and 17 individuals with RTS. Both participant groups also showed a similar pattern of spontaneous visual preference for emotions.

**Conclusions:**

These results provide insight into two rare, genetically defined neurodevelopmental disorders that have been reported to exhibit contrasting socio-behavioural characteristics and suggest that differences in social behaviour may not be sufficient to predict attention to the eye region of faces. These results also suggest that differences in the social behaviours of these two groups may be cognitively mediated rather than subcortically mediated.

## Background

The processing of social information is crucial for understanding the social world in which we live. In order to identify people during social interactions, we must process their facial features and characteristics. Furthermore, information gained from the face, such as expressions of emotion, can inform whether it is necessary to alter our interaction style. Exploring the face to spontaneously distinguish emotional expressions is part of successful social interaction. The eye region, in particular, has been proposed to be highly important for social interaction, due to the role it plays in conveying emotional states and communicative intent [1, 2].

Different face scanning has been reported in the literature for individuals who exhibit impairments in social interaction [3–5]. However, the majority of these studies have focussed primarily on individuals with autism spectrum disorder (ASD), although more recent studies have investigated visual exploration of social stimuli in Williams syndrome (WS). These two neurodevelopmental disorders are each associated with impairments and atypicalities in social interactions, but the presentation of these impairments is dramatically different. For example, individuals with ASD have often been reported to exhibit social withdrawal and reduced eye contact, whereas individuals with WS have been reported to exhibit hyper-sociability and heightened eye gaze [3–5].

In addition to distinctive patterns of eye looking in individuals with neurodevelopmental disorders associated with divergent socio-behavioural characteristics, reduced eye looking has been well documented in individuals with amygdala damage. For example, failure to spontaneously fixate to the eye region of static faces has been reported in a patient with bilateral amygdala damage [6]. Reduced eye contact during real social interactions has also been shown in a patient with amygdala damage [7], and a positive relationship between amygdala activation and looking to the eye region of faces has been documented in ASD [8]. Although evidence for amygdala dysfunction in ASD exists, it is somewhat inconsistent [9, 10]. In addition, reduced eye looking in ASD has been proven to be more inconsistent than once thought. Indeed, many studies have reported no difference in the amount of time individuals spend looking at the eye region compared to typically developing controls [11–13]. Rather, reduced eye looking in ASD has recently become most commonly associated with dynamic stimuli as opposed to static stimuli [12]. This suggests that reduced eye looking in ASD may be mediated by higher order cognitive mechanisms as opposed to biologically mediated amygdala dysfunction, with which reduced eye looking to static faces is a more consistent finding than in ASD.

It has been suggested that impaired facial emotion recognition may be due to reduced looking at the eye region, which has been argued to be important for communicating emotional expressions [2, 14, 15]. In support of this, eye contact has been reported to predict performance on a facial emotion recognition task in individuals with ASD [14], and increased emotion recognition performance has also been reported in those looking longer to the eye region [15]. Furthermore, neuropsychological patients with damage to the amygdala have been found to exhibit both reduced looking to the eye region of static faces and reduced ability to discriminate facial expressions of emotion [6, 16]. Both of these findings are in line with the hypothesis that looking to the eye region is important for successful emotion recognition, which in turn has been suggested to be important for successful social interaction [17]. However, more recent studies have also suggested that the eye region may not be as crucial as once thought, with a recent study showing reduced eye contact in a group of participants with ASD who also displayed intact emotion recognition skills [18]. Furthermore, a number of studies have reported relatively intact emotional face processing in ASD when comparison groups are well matched [19], thus highlighting the mixed nature of the findings regarding emotion recognition and looking to the eye region in ASD.

The aforementioned studies have revealed a putative pathway from eye gaze behaviour during the viewing of social stimuli and social characteristics in ASD versus WS [3–5]. Whilst these studies report clear findings that reflect the characteristic social behaviours of the groups studied, the two disorders are also associated with socio-behavioural profiles argued to be at polar ends of a spectrum [20]. Whether or not similar group level or individual associations are replicable with different neurodevelopmental disorders associated with contrasting socio-behavioural profiles has not yet been investigated. Cornelia de Lange syndrome (CdLS) and Rubinstein-Taybi syndrome (RTS) show a divergent pattern of social abilities. Whether or not the social behaviours exhibited by individuals with CdLS and RTS, namely social withdrawal/anxiety and social interest, can be linked to visual exploration of social information in the same was as previously reported in ASD and WS is of interest to the present study.

CdLS is a genetic disorder affecting approximately 1 in 40,000 live births [21] and is associated with intellectual disability, specific physical characteristics such as distinctive facial features and limb abnormalities and increased rates of ASD symptomatology [22–24]. CdLS is primarily caused by a deletion in the NIPBL gene located on chromosome 5 [25–27], whilst fewer cases have been reported that are caused by mutations on the SMC3 gene on chromosome 10 [28], the SMC1 gene [29], the HDAC8 gene [30], and the RAD21 gene [31].

Although CdLS is associated with an increased prevalence of ASD, the social impairments are subtly different. Most notably, individuals with CdLS have been reported to exhibit extreme social anxiety alongside selective mutism, whereas those with ASD typically withdraw from social interaction, but this withdrawal is not commonly or primarily attributed to extreme anxiety [24]. In addition, individuals with CdLS have been reported to exhibit reduced eye contact during situations which require initiation of speech [32] but less impaired eye contact than individuals with ASD [24]. One possible explanation for this pattern of eye looking in CdLS is that decreased eye contact is associated with anxiety and social withdrawal tendencies during situations with high social demand, whereas less impaired eye contact during a range of situations may be reflective of relatively higher social motivation in this population [24]. Interestingly, increased eye contact is also reported in typically developing individuals experiencing social anxiety [33]. Furthermore, a role for amygdala dysfunction in social anxiety has been postulated [34, 35].

The social impairments documented in CdLS are not dissimilar to those seen in fragile X syndrome (FXS), with both groups generally being reported or suggested to exhibit heightened social anxiety alongside heightened social motivation [36]. Eye-tracking methodology has previously been used to investigate face scanning in individuals with FXS, with the majority of these studies reporting reduced looking to the eye region in comparison to typically developing individuals [37–40] and in comparison to children with ASD [41]. Interestingly, FXS is associated with amygdala dysfunction [42], which, with consistent reports of reduced looking to the eye region of faces, may indicate that the social impairments observed in this group are somewhat subcortically mediated by amygdala dysfunction. Furthermore, direct comparisons of the brain structure in individuals with FXS and ASD have revealed smaller amygdala size in those with FXS compared to those with ASD [10]. This provides further support for the notion that eye looking in ASD may be mediated by higher order cognitive mechanisms as opposed to biologically mediated by amygdala dysfunction, as is more likely to be the case in FXS.

Whilst eye contact has been investigated in real-life social situations in people with CdLS, the use of eye-tracking methodology to investigate face scanning has not previously been attempted. Investigating the visual exploration of social information such as faces and emotional expressions using robust methodological techniques is important due to the relationship this may have to the striking social impairments present in this group. Furthermore, brain-imaging studies have not been conducted in CdLS. Therefore, studying eye looking and emotion processing, which has been associated with amygdala damage, may further our understanding of amygdala function in an under-researched population.

RTS is also a genetic syndrome associated with intellectual disability, affecting approximately one in 100,000–125,000 live births [43]. Mutations in the CREB-binding protein gene (CBP) account for approximately 40 % of cases, whereas mutations in the *EP300* gene account for a limited number of cases [44–48]. Whilst studies investigating the social characteristics of RTS are limited, those that have been conducted to date suggest that social skills are largely intact in this group relative to their level of intellectual functioning [49]. Individuals with RTS have been reported to initiate and maintain social contact despite cognitive impairments [47]. One study, for example, described three children with RTS as being friendly and as making good social contacts [50]. Families of children with RTS have also described them as friendly and loving [50–52]. Increased social interest in children with RTS particularly relating to eye contact, initiating play, and use of facial expressions, compared to a group of children matched for age, gender, and developmental ability [53] has also been reported. However, this report of increased social interest given the level of intellectual ability may be age-specific, as an increase in anxiety and depression in adults with RTS compared to children with RTS has recently been reported [54]. However, as the majority of research indicates typical levels of social interest in this group, if not increased, it would be interesting to investigate the visual exploration of social information in this syndrome group. Similarly to CdLS, eye-tracking methodology has not yet been used to investigate looking patterns to social stimuli in this group.

The current study uses eye-tracking methodology to investigate spontaneous emotion preference for happy versus neutral and disgust versus neutral facial expressions and face scanning in individuals with CdLS versus RTS. Happiness and disgust were the expressions used in the present study due to their contrast in emotional valence. Many negative emotional expressions, such as sadness, fear, and anger, can often be experienced cognitively with no distinctive facial expression. For example, one may not always display a frown when experiencing sadness. Disgust was chosen as the negative emotional expression of interest for the current study as it is depicted facially. Patterns of eye gaze across the eye, mouth, and other regions of the face were also measured during “standard” trials, which presented pairs of faces posed in neutral expressions, in order to examine and compare gaze to the eye region across participant groups.

The aim of this study is to determine whether or not previous findings in individuals with contrasting profiles of social behaviour, namely ASD (reduced) and WS (enhanced), replicate in the visual exploration of social information of two syndrome groups that exhibit similarly contrasting socio-behavioural characteristics. As impaired eye looking in static faces is associated with amygdala dysfunction, this study aims to further the understanding of whether the documented differences in social behaviour of CdLS and RTS are subcortically or cognitively mediated. Based on previous literature indicating differences in face processing for groups with divergent profiles of social behaviour, we hypothesised that those with CdLS and RTS would show contrasting patterns of looking to the eye region. Specifically, based on reports of heightened social anxiety in people with CdLS [24, 32] and reports of social interest being relatively intact in individuals with RTS [53], we predicted that individuals with CdLS would exhibit less looking to the eye region than individuals with RTS.

## Methods^1^

### Participants

Fifteen individuals with CdLS (seven female, M_age_ = 18.42, SD = 9.78) and 17 individuals with RTS (10 female, M_age_ = 17.33, SD = 10.14) were included in the analyses. An additional one participant with RTS was tested but did not provide reliable data due to providing over 40 % invalid trials in one condition. A trial during which the participant did not look at either face was considered invalid. Table 1 presents the characteristics of the final study populations. As Table 1 shows, participants with CdLS and RTS were matched on chronological age, gender, severity of autistic impairments, as measured by the social communication questionnaire (SCQ; [55]), and global and communicative adaptive behaviour abilities, as measured by the Vineland adaptive behaviour scale (VABS; [56]). SCQ data was not returned for one participant with RTS. Participants were recruited through the Cerebra Centre for Neurodevelopmental Disorders, University of Birmingham (UoB) participant database, through the Cornelia de Lange Foundation UK and Ireland, and through the Rubinstein-Taybi syndrome UK support group. All participants had a confirmed diagnosis from a professional (paediatrician, general practitioner, or clinical geneticist). Two participants with CdLS were tested at the UoB. All remaining participants were tested at syndrome support group family meetings. This study was reviewed and approved by the School of Psychology Ethics Committee at the UoB. Written consent was obtained from participants aged 16 years and over and parents of children under 16 years of age before their participation in the study.

**Table 1:** Participant characteristics and alpha level for comparison between CdLS and RTS participants

Characteristic	CdLS	RTS	*P*
	(*n* = 15)	(*n* = 17)	
Mean age in years (SD)	18.42 (9.78)	17.33 (10.14)	0.760
Age range in years	6.70–33.36	4.31–37.10	NA
Gender (% male)	53.33	41.18	0.492
Mean adaptive behaviour composite (SD)	59.87 (24.99)	58.53 (15.08)	0.854
Adaptive behaviour composite range	20–121	30–84	
Mean communication standard score (SD)	53.47 (25.89)	59.00 (20.16)	0.503
Communication standard score range	21–104	21–92	
Mean SCQ (SD)	13.84 (4.34)	14.01 (4.14)	0.914
SCQ total score range	5–22	8–20	NA
Participants meeting SCQ cutoff for ASD (%)	4 (26.67)	7 (43.75)	0.387

Comparison between participants on chronological age, gender, adaptive behaviour composite, and communication subscale standard scores as measured by the Vineland adaptive behaviour scale—survey form, mean score, and number of participants meeting ASD cutoff on the social communication questionnaire (SCQ)

### Apparatus

The experimental procedures described here are the same as those used by the authors in a previous study, which reported a difference in looking times to the eye region of facial stimuli in individuals with FXS and ASD [41]. The stimuli were generated by the Experiment Builder programme (SR Research, Ontario, Canada) and presented on a 19-in. CRT screen at a screen resolution of 1024 × 768. Participants placed their head on a chin rest 0.6 m from the screen, in a dimly lit room with windows blacked-out to avoid luminance changes. Chin rest and desk heights were adjusted so that eye gaze was central to the display screen. Eye movements were recorded using an Eyelink 1000 Tower Mount system, which runs with a spatial accuracy of 0.5–1 visual angle (°), a spatial resolution of 0.01°, and a temporal resolution of 2 (500 Hz). A five-point calibration was performed prior to each experimental block, as well as mid-block if necessary. A single-point drift correction to the calibration was made prior to every fifth trial. The eye-tracking camera was linked to a separate host PC to the one displaying the search stimuli. EyeLink software (SR research, Ontario, Canada) was used to control the camera and collect data and was synchronised via an Ethernet cable with display PC.

### Stimuli

During the eye-tracking task, an animated dolphin measuring 0.96 × 1.43° of visual angle was used for calibration, as well as for drift correction and fixation “cross” prior to each trial. The 38 static colour photographs of male and female adult human faces were taken from the MacBrain Face Stimulus Set^2^ [57]. During each trial, two faces were presented side-by-side. On the majority of trials, both faces displayed a neutral facial expression. For the remainder of trials, one of the two faces displayed a happy or disgusted expression. The faces displayed a straight-ahead gaze and an open mouth. Only the face, hair, and neck were visible. Faces subtended an average of 14.30 × 18.59° of visual angle were displayed on a white background. They were positioned side-by-side, separated by a gap of 7.179° of visual angle.

### Measures

In addition to participants completing the eye-tracking task, the participant’s primary caregiver completed a demographic questionnaire providing information about the participants’ gender, date of birth, verbal ability (more/less than 30 signs/words), and mobility (ability to walk unaided). Information about the participant’s diagnosis was also collected from caregivers including the specific diagnosis given, who gave the diagnosis, and when. Participants’ primary caregivers also completed the SCQ [55], to assess behaviours associated with ASD such as social functioning and communication skills. A score of 15 or above is suggested by the authors of the SCQ to indicate the presence of an ASD. The Communication Skills, Daily Living Skills, and Socialisation Skills of the Vineland Adaptive Behaviour Scale—Second Edition, Survey Interview Form [56] was administered to primary caregivers to assess participants’ adaptive behaviour abilities. The interview yields an adaptive behaviour composite (ABC) from the three domains. Standard scores, which are based on a sample of 3000 children, can be calculated for each domain and the ABC and reflect performance relative to participant chronological age. The standard scores for the communication domain and the ABC were used in the present study to ensure participants were matched on communicative and global adaptive behaviour abilities.

### Eye-tracking task

All participants were instructed to remain still during testing. At the start of the eye-tracking task, the eye-tracker was calibrated using a five-point calibration. During calibration, participants fixated on an animated blue dolphin as it moved positions from the centre of the screen to various locations around the edges of the display area. The calibration was repeated until all participants achieved a full five-point calibration. In between each trial, the animated dolphin reappeared at the centre of the screen to act as a point of fixation. Every five trials, this individually presented dolphin served as a one-point drift correct to adjust calibration of the eye-tracker accounting for small head movements. If necessary, re-calibration was undertaken at this point and the trials resumed once calibration was successful.

Participants were presented with 80 trials, during which two faces were presented side-by-side for 1500 ms. The animated dolphin was displayed for 1000 ms in between trials, except for trials when a drift correct was performed. This was a passive viewing task. Therefore, participants were instructed to look wherever they wished whilst the faces were presented on the screen but to look at the dolphin that appeared between trials. Participants completed one of two experimental blocks, each with trials in a different pseudo-random trial presentation order. As a result of randomization, in one experimental block, 10 of 80 trials were “emotion” trials in which one emotionally expressive face was presented alongside one neutrally expressive face; in the other experimental block, 11 of 80 trials were “emotion” trials. The experimental block assigned to participants was counterbalanced within and across participant groups. The remaining trials were “standard” trials, in which two neutrally expressive faces were presented in order to habituate participants to the category of neutral facial expressions. To ensure participant’s habituation to neutrally expressive faces, the beginning of the testing block commenced with at least seven “standard” trials prior to the presentation of any “emotion” trials. Throughout the remainder of the experiment, “emotion” trials were separated by a minimum of four “standard” trials. During “emotion” trials, the emotionally expressive face displayed either happiness or disgust and was equally likely to appear on the left or right side of the screen. Happy faces were presented during approximately half of the emotion trials in both experimental blocks. Disgust was presented during the remainder of the emotion trials. The eye-tracking task generally lasted less than 10 min but total experiment time varied slightly across participants due to differences in the amount of time it took to obtain successful calibration and whether participants accepted the option to take additional breaks during the drift-correct trials.

### Procedure

Participants completed the eye-tracking task, and parents of participants completed the SCQ and the VABS. The eye-tracking task was completed first. Parents completed the SCQ either whilst their child performed the eye-tracking task or at home and returned it to the researchers. The VABS was either administered face to face following the eye-tracking task or over the telephone following the testing session.

### Data analysis

**Fig. 1:**
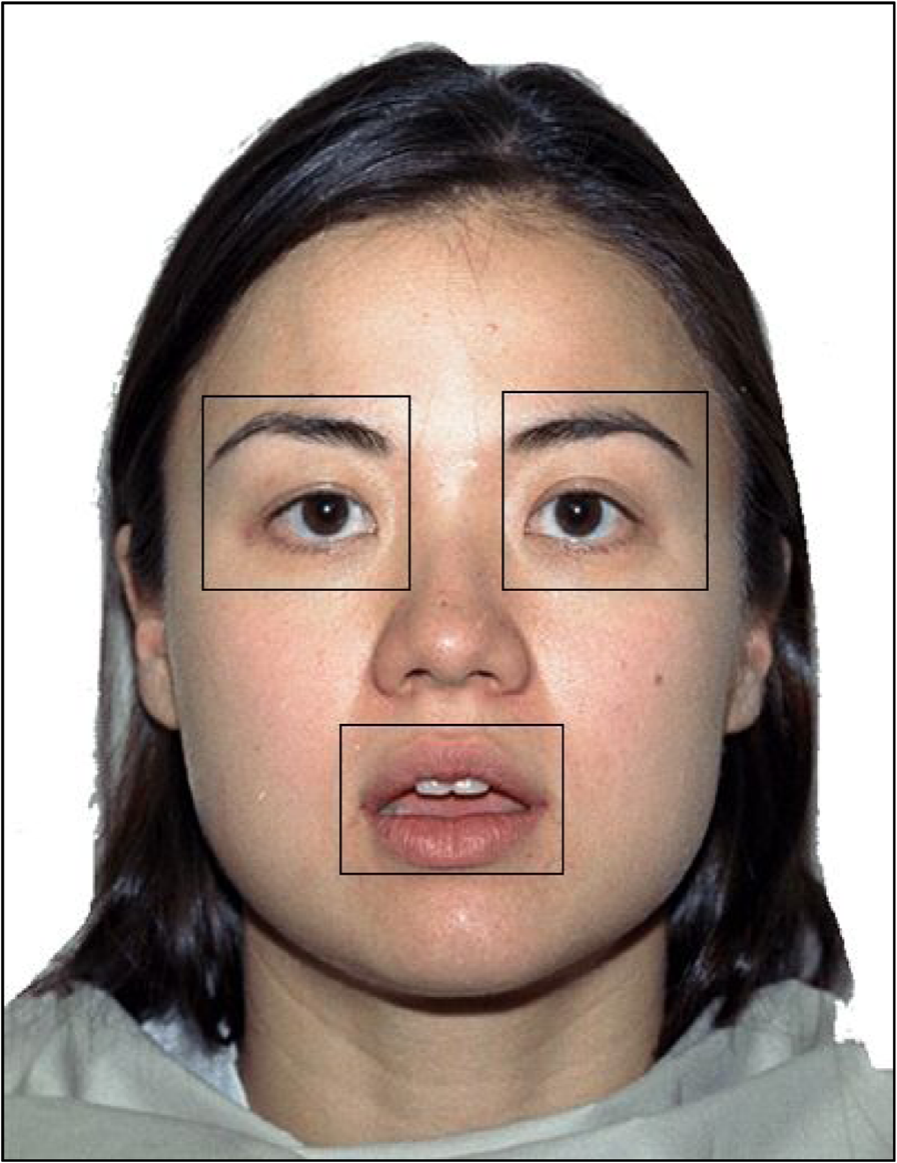
Example of face ROI, left and right eyes ROI, and mouth ROI; face ROI was a rectangular shape positioned automatically to cover the face, hair, and neck of models, whilst fixation coordinates within the rectangular areas were assigned to eyes and mouth ROI for each model, respectively

Fixations were assessed as occurring when eye movement did not exceed a velocity threshold of 30°/s, an acceleration threshold of 8000°/s^2^, or a motion threshold of 0.1°, and the pupil was not missing for three or more samples in a sequence. A fixation was assigned to a particular area of the face when the fixation coordinates were within a rectangular area (termed the “region(s) of interest” or ROI) assigned to the area in question. Face ROI was a rectangular shape positioned automatically to cover the face, hair, and neck of the models presented on the left and right side of the screen, whilst ROI for the left eye, right eye, and mouth for each individual face were identified manually using coordinates (see Fig. 1). The ROI for all stimuli were identical to those previously reported in a study using the same paradigm to investigate eye looking and emotion preference in FXS and ASD [41]. All data were subjected to the Shapiro-Wilk test for normality.^3^ The mean number of trials with missing data (where participants did not look at either facial stimulus) was 4.133 for participants with CdLS and 4.82 for participants with RTS. Except where mentioned, the alpha level for significance was 0.05.

## Results

Participants with CdLS spent, on average, 37.8 % of trial time looking at the facial stimuli and 25.4 % of trial time looking at other areas on the screen. This was similar for participants with RTS as they spent 38.5 % of trial time looking at the facial stimuli and 25.9 % of trial time looking at other areas on the screen. On average, data from 35.8 and 35.6 % of trial time from participants with CdLS and RTS, respectively, were lost due to saccades, blinks, and inattention. Previously published data from typically developing children and adults [41], who completed the same paradigm, yielded similar percentages of lost data (41.4 and 34.3 %, respectively). Spontaneous emotion preference data are presented as proportion of trial spent looking, in seconds, at faces posed in happy, disgust, and neutral facial expressions. Eyes and mouth looking data were only analysed during standard trials, on which both faces presented neutral expressions. Eye looking data are presented as a ratio of the time spent looking at the eyes to the time spent looking at the face:

$$ \frac{\mathrm{Mean}\ \mathrm{time}\ \left(\mathrm{in}\ \mathrm{ms}\right)\ \mathrm{spent}\ \mathrm{looking}\ \mathrm{at}\ \mathrm{the}\ \mathrm{left}\ \mathrm{eye} + \mathrm{mean}\ \mathrm{time}\ \left(\mathrm{in}\ \mathrm{ms}\right)\ \mathrm{spent}\ \mathrm{looking}\ \mathrm{at}\ \mathrm{right}\ \mathrm{eye}}{\mathrm{Mean}\ \mathrm{time}\ \left(\mathrm{in}\ \mathrm{ms}\right)\ \mathrm{spent}\ \mathrm{looking}\ \mathrm{at}\ \mathrm{neutral}\ \mathrm{faces}} $$

Mouth looking data are presented as a ratio of the time spent looking at the mouth to the time spent looking at the face:

$$ \frac{\mathrm{Mean}\ \mathrm{time}\ \left(\mathrm{in}\ \mathrm{ms}\right)\ \mathrm{spent}\ \mathrm{looking}\ \mathrm{at}\ \mathrm{the}\ \mathrm{mouth}\ \mathrm{region}}{\mathrm{Mean}\ \mathrm{time}\ \Big(\mathrm{in}\ \mathrm{ms}\ \mathrm{spent}\ \mathrm{looking}\ \mathrm{at}\ \mathrm{neutral}\ \mathrm{faces}} $$

There were no between-group differences in the amount of time spent looking at the screen (*t*(30) = −0.639, *p* = 0.528) or in the amount of time participants spent looking at faces relative to the background of the screen (*t*(30) = 0.538, *p* = 0.594).

### Eyes/mouth looking time

Data reflect the amount of time in milliseconds that was spent looking at the left eye ROI, the right eye ROI, and the mouth ROI. In order to account for different looking time on faces, the average time each participant spent looking at the eyes and mouth of the neutral faces presented during standard trials was divided by the average amount of time that participant spent looking at both neutral faces. Emotional face (i.e. oddball) trials were not included in these analyses due to the low percentages of trials that they represented, as well as the fact that participant looking time was split between neutral and emotional faces on these trials.

**Fig. 2:**
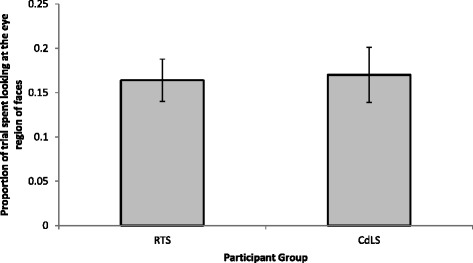
The amount of time spent looking at the eye region of neutral faces; the amount of time, in milliseconds, spent looking within the eyes ROI divided by the amount of time, in milliseconds, spent looking at the entire face ROI of neutral faces. *Error bars* represent standard error

To ensure that participants did not demonstrate a looking bias to the left or right eye in faces, *t* tests were conducted for each group to compare looking time to the left and right eyes relative to the amount of time spent looking at the face which revealed no significant differences (CdLS: *t*(14) = 0.557, *p* = 0.586; RTS: *t*(16) = −1.759, *p* = 0.098). Therefore, the time spent looking to the left and right eye, relative to the amount of time spent looking at faces, was summed for further analyses in order to investigate overall looking patterns to the eyes. Figure 2 depicts the ratio of time each group spent looking at the eye region of faces.

To compare looking time to the eye region of the faces, an independent sample *t* test was conducted. The analysis revealed no significant between-group difference in the ratio of time spent looking at the eyes to the time spent looking at faces (*t*(30) = −0.158, *p* = 0.875).

**Fig. 3:**
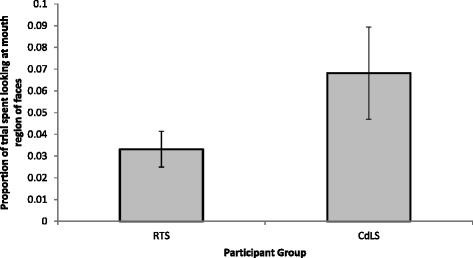
The amount of time spent looking at the mouth region of neutral faces; the amount of time, in milliseconds, spent looking within the mouth ROI divided by the amount of time, in milliseconds, spent looking at the entire face ROI of neutral faces. *Error bars* represent standard error

**Fig. 4:**
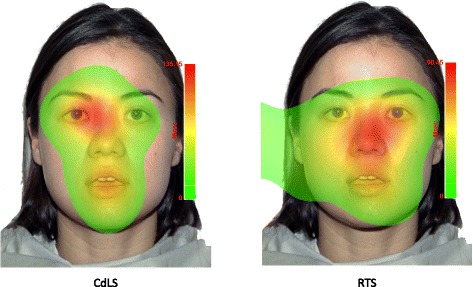
Heat maps depicting the distribution of looking on all neutral trials. The heat map is based on the duration of fixations across the display for all participants. The eyes and mouth were not exactly lined up across all trials due to natural variation in the position of features across the different facial stimuli

In order to compare looking time to the mouth region of the faces relative to the rest of the face, an independent sample *t* test was conducted. The analysis revealed no significant between-group difference in the ratio of time spent looking at the mouth (*t*(18) = −1.537, *p* = 0.142). Figure 3 depicts the ratio of time each group spent looking at the mouth to the rest of the face. Due to the wide range of ages and abilities of participants included in this study, an analysis of covariance (ANCOVA) was conducted, which revealed no effect of syndrome group on the amount of time spent looking at the eyes or mouth relative to the amount of time spent looking at the face, when chronological age was controlled for (eye looking: *F* (1, 29) = 0.038, *p* = .846; mouth looking: *F* (1, 29) = 2.505, *p* = 0.124) and when global adaptive behaviour ability was controlled for (eye looking: *F* (1, 29) = 0.017, *p* = 0.896; mouth looking: *F* (1, 29) = 2.613, *p* = 0.117). Figure 4 presents the heat maps for each participant group to depict the distribution and duration of looking to neutral faces.

### Spontaneous emotion preference

The proportion of the trial spent looking at faces displaying a happy expression was calculated for happy faces and neutral faces presented side-by-side with happy faces. This process was repeated for dwell time percentage on faces displaying a disgusted expression and for neutral faces presented alongside disgusted faces. Paired sample *t* tests were conducted for each group to investigate whether participants spent a significantly higher proportion of the trial looking at happy relative to neutral faces during happy-neutral trials and disgust relative to neutral faces during disgust-neutral trials. These *t* tests revealed that both participant groups spent a higher proportion of the trial looking at disgust compared to neutral faces (CdLS: *t*(14) = 2.761, *p* = 0.015; RTS: *t*(16) = 5.997, *p* < 0.001) but not happy compared to neutral faces (CdLS: *t*(14) = 0.617, *p* = 0.547; RTS: *t*(16) = 0.799, *p* = 0.436).

**Fig. 5:**
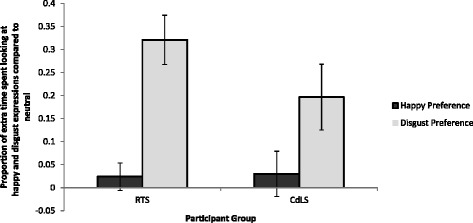
Looking preference for happy and disgust faces, compared to neutral faces; the proportion of trial time that participants spent looking at happy faces divided by neutral faces during happy-neutral trials (happy preference), and the proportion of trial time that participants spent looking at disgusted faces divided by neutral faces during disgust-neutral trails (disgust preference). *Error bars* represent standard error

The analysis conducted thus far indicated that both participant groups look more at disgust faces than neutral faces but not happy faces compared to neutral faces. However, this analysis does not allow for a between-group comparison. Therefore, a looking preference for happy faces was calculated by subtracting the proportion of the trial spent looking at neutral faces during happy-neutral trials from the proportion of the trial spent looking at happy faces. This was repeated to calculate the disgust preference. Happy and disgust preferences were compared between groups using an independent samples *t* test. This test indicated no between-group difference of happy preference (*t*(30) = −0.115, *p* = 0.909) or disgust preference (*t*(30) = 1.414, *p =* 0.168). Figure 5 depicts the proportion of extra time spent looking at happy and disgust faces compared to neutral faces during oddball trials. In summary, all participants spent a higher proportion of time looking at disgust versus neutral faces but not happy versus neutral faces. Due to the wide range of ages and abilities of participants included in this study, an ANCOVA was conducted, which revealed no effect of syndrome group on happy or disgust preference, when chronological age was controlled for (happy preference: *F* (1, 29) = 0.009, *p* = 0.924; disgust preference: *F* (1, 29) = 1.941, *p* = 0.174) or when global adaptive behaviour was controlled for (happy preference: *F* (1, 29) = 0.028, *p* = 0.868; disgust preference: *F* (1, 29) = 1.923, *p* = 0.176.

## Discussion

In the present study, we investigated looking patterns to the eyes and mouth, as well as spontaneous emotion preference, in individuals with CdLS and RTS. In line with previous literature that provides evidence for different patterns of visual exploration of social stimuli in groups displaying divergent social behaviours, it was hypothesised that individuals with CdLS and RTS would also demonstrate different patterns of face scanning due to their contrasting socio-behavioural profiles. Specifically, we predicted that individuals with CdLS would display lower levels of looking to the eye region than those with RTS, due to the reports of social anxiety and withdrawal reported in CdLS [32] and due to the heightened social interest reported in RTS [53]. The results demonstrate that individuals with CdLS and RTS displayed similar looking patterns to the eye region of the face. These findings do not support the hypothesis of a difference between groups with contrasting profiles of social behaviour exhibiting different face processing techniques. Furthermore, as existing literature points to a role for amygdala dysfunction in reduced looking to the eye region of static faces [6], the results from the present study indicate that the documented differences in CdLS and RTS are unlikely to be subcortically mediated.

Spontaneous looking patterns were assessed by examining and comparing the ratio of time spent looking at the eyes and mouth during the standard trials (neutral face pairs). The results indicated that participants with CdLS and RTS looked at the eye region of the faces a similar amount. These findings are unlikely to be driven by chronological age, autistic impairments, and global and adaptive behaviour ability levels as these variables were matched across participants. Whilst the expected group differences between CdLS and RTS did not emerge, it is unlikely that the lack of group differences in the present study is a result of the paradigm used and its sensitivity to highlight group differences. Using the same paradigm, the authors previously reported that participants with FXS exhibit reduced looking to the eye region of the faces, in comparison to those with ASD, as used here [41]. Although previously published data from participants with ASD and TD children and adults [41] were not presented here, those data were compared to data presented for participants with CdLS and RTS in the current study. Interestingly, no differences were found between any groups suggesting typical eye gaze in both those with CdLS and RTS. Reduced eye looking in FXS compared to ASD using the same measure lend support to the notion that the social impairments in FXS are somewhat subcortically mediated by amygdala dysfunction, which has been reported in this population [42]. However, amygdala dysfunction is a less consistent finding in ASD [9] and may go some way toward explaining inconsistent results regarding looking to the eye region of faces [12]. In the present study, no differences in looking to the eye region between individuals with CdLS, associated with social withdrawal and anxiety, and individuals with RTS, associated with social interest, were found. These results indicate that firstly, the documented differences in social behaviour in CdLS and RTS may not be subcortically mediated. Consequently, this suggests that the social anxiety reported in CdLS may be cognitively mediated, rather than associated with amygdala dysfunction, which has implications for both basic science and clinical intervention in relation to individuals with CdLS.

Previous literature comparing visual exploration of social stimuli in ASD and WS has consistently reported less eye looking in ASD, associated with social withdrawal, and increased eye looking in WS associated with hyper-sociability [3–5]. However, the present study reports similar eye gaze patterns in two different neurodevelopmental disorders also associated with clearly contrasting profiles of social behaviour. From previous reports of social behaviour in CdLS and RTS appearing to differ and from previous studies of face scanning in disorders with contrasting socio-behavioural characteristics, differing levels of eye gaze were predicted in these groups. However, such differences were not observed in the present study, and both groups showed typical levels of eye gaze. One possible explanation for these results concerns the nature of the differences in social skills between those with CdLS and those with RTS. Whilst there are documented differences in the social behaviours of the two disorders studied here, the differences are perhaps not as extreme as those described in ASD and WS, arguably at polar ends of a sociability spectrum. The lack of a clear distinction of visual exploration of social stimuli in the current groups, whose associated socio-behavioural characteristics are contrasting, suggests that studying social cognition across individuals with different genetic syndromes and neurodevelopmental disorders is often more complicated than the impaired or enhanced profile of results that emerge from those with ASD and WS. The results from this study suggest that clear differences in socio-behavioural characteristics are not sufficient to predict attention to social information. Furthermore, whilst eye contact has been reported to be a good indicator of social functioning in ASD and WS, this may not be the case for all neurodevelopmental disorders. Existing evidence exists to support this interpretation. Specifically, some studies have reported a developmental shift in the relationship between reduced eye looking and social disability. For example, reduced eye looking has been associated with higher levels of social disability in toddlers [58, 59], but there appears to be no such relationship in school-age children [60] or adults with ASD [61]. The mean age of participants in the present study was 17–18 years. Therefore, it may be the case that visual attention to social information may be more predictive of socio-behavioural impairments in the early years of life as opposed to throughout early adulthood.

It is important to consider the interpretation of these findings in light of the limitations of the study. Firstly, behavioural data on these two participant groups were not collected alongside the eye-tracking data. Although it is common in the existing literature for data to be presented on either behavioural or cognitive measures, previous studies documenting looking patterns to social stimuli have previously used participant groups with well-defined socio-behavioural characteristics, such as ASD and Williams syndrome. As the social behaviour of CdLS and RTS is comparatively under-researched, it would have been beneficial to collect such data on the individuals participating in the present study and this is a focus for future research. Instead, for the present study it was necessary to utilise previous literature to document the socio-behavioural characteristics of CdLS and RTS, and interpret the current results in light of existing literature. Whilst the sample size is comparable to existing studies investigating visual attention to social stimuli in genetic syndromes versus typical development, there may be limited power in the current study to detect smaller differences between two participant groups where different socio-behavioural features are documented yet require more extensive exploration. In addition, the conclusions stated here should be considered alongside the potential limitation that this study documents eye looking during a laboratory-based task of passive viewing of facial stimuli, which, whilst providing robust and novel findings, does not mirror real-world experiences of social interactions. Due to the laboratory-based setting, the facial stimuli presented may be less anxiety provoking than real faces, which could impact the way in which they are processed. Future research should consider the differential effects of laboratory and real-world experiences in visual exploration of faces in children and adults with neurodevelopmental disorders. Finally, whilst IQ measures were not administered for the present study, the VABS adaptive behaviour composite and communication standard score provide standard and reliable measures of adaptive behaviour abilities and verbal abilities, respectively, that are comparable across the CdLS and RTS groups. Adaptive behaviour was assessed over IQ measures due to the difficulty associated with selecting an IQ test that can be administered to individuals with a range of chronological ages and abilities. In addition, due to the level of intellectual ability of participants in this study, it was deemed more appropriate to use parental report of adaptive behaviour abilities, which focus on typical performance of everyday skills, as opposed to IQ measures, which focus on optimal performance of tasks that are associated with performance or cognitive demands. Furthermore, general IQ has been reported to correlate with the communication subscale of the VABS [62, 63], which did not differ between the CdLS and RTS participant groups in the current study. It should be noted that, to our knowledge, this is the first study documenting the use of eye-tracking technology in individuals with CdLS and RTS. Due to the level of intellectual disability associated with these genetic syndromes, the use of a passive viewing task was deemed most appropriate. Importantly, the overall levels of task engagement reflect those demonstrated by TD children and adults on the same paradigm. Therefore, these levels of task engagement, which may be considered relatively low, most likely reflect the nature of the task used. As passive viewing tasks do not require a response, there is no cost to the participant to look away from the screen.

In addition to the findings on face scanning, the results from the current study showed that implicit emotion preference did not differ in either individuals with CdLS or individuals with RTS. In the current study, spontaneous emotion preference was assessed using a novel oddball paradigm in conjunction with a preferential looking measure. Participants were presented with pairs of neutral faces (standard trials), with neutral-disgust, and neutral-happy pairs (oddball trials) presented infrequently. Participants in both groups looked longer at faces posed in disgusted expressions compared with neutral faces during the target trials, whereas no participant group looked longer at the faces posed in happy expressions compared to neutral faces. This pattern of results mirrors those previously reported for TD children and adults [41].

As described above, participants in both groups exhibited significant preferential looking to disgust relative to neutral expressions but did not exhibit a preference for looking to happy relative to neutral expressions. Two potential explanations for these findings are proposed. Firstly, it is possible that disgusted faced gain an attentional advantage over happy faces due to the relative novelty of disgusted faces (see [64] for a review). Disgusted faces are not seen in everyday life as often as happy faces. Therefore, the novelty of the disgusted faces may have captured the attention of participants to a greater extent than the happy faces. Alternatively, the negativity bias, whereby individuals attend more to negative information than to positive information due to its increased *informational* value in detecting threatening stimuli [65, 66], may also contribute to the results reported in the current study. Disgusted expressions may be perceived as a cue to threat, due to its association with negative affect, thus capturing an individual’s attention more so than non-threatening, positive facial expressions.

## Conclusions

The results of this study show similar face scanning in two neurodevelopmental disorders with contrasting profiles of social behaviour. Individuals with CdLS and RTS looked at the eye region of faces a similar amount. Spontaneous emotion preference was also observed to be similar in those with CdLS and RTS in the current study and mirror that previously reported in TD individuals [41]. These findings suggest that such coarse measures as attention to the eyes may not be sensitive to differences in socio-behavioural characteristics unless the differences are as extreme as those seen in ASD and WS. These findings also suggest that documented differences in the socio-behavioural characteristics of individuals with CdLS and RTS may be cognitively rather than subcortically mediated, due to the well-documented association between impaired eye looking and amygdala dysfunction. Future experimental eye-tracking and other research should focus on other aspects of social cognitive functioning and social behaviour in individuals with CdLS, RTS, and other genetic syndromes, in an effort to elucidate pathways from genetic disorders to behaviour in atypical and impaired social functioning.

## Abbreviations

ABC: adaptive behaviour composite
ASD: autism spectrum disorder
CdLS: Cornelia de Lange syndrome
FXS: fragile X syndrome
RTS: Rubinstein-Taybi syndrome
SCQ: social communication questionnaire
TD: typically developing
UoB: University of Birmingham
VABS: Vineland adaptive behaviour scale
WS: Williams syndrome

## Footnotes

1 The methods used here are identical to those used and described in a previous study conducted by the authors to investigate face processing in Fragile X Syndrome and Autism Spectrum Disorder [41].

2 Development of the MacBrain Face Stimulus Set was overseen by Nim Tottenham and supported by the John D. and Catherine T. MacArthur Foundation Research Network on Early Experience and Brain Development. Please contact Nim Tottenham at tott0006@tc.umn.edu for more information concerning the stimulus set.

3 Where results from non-parametric tests, used when data were not normally distributed, did not differ from results from the equivalent parametric tests, the results from the parametric tests are reported.
